# Comparative Evaluation of Electron Paramagnetic Resonance and Spectrophotometry for DPPH-Based Antioxidant Capacity Determination

**DOI:** 10.1021/acsomega.6c03248

**Published:** 2026-09-22

**Authors:** Helvis L. S. Fontoura, Ana Flávia L. M. Nascimento, Francine A. F. Menezes, Pâmela V. Rocha, Stanislau Bogusz, Nádia R. Pereira, André O. Guimarães

**Affiliations:** † 28109 Universidade Estadual do Norte Fluminense Darcy Ribeiro, Laboratório de Ciências Físicas, Campos do Goytacazes 28.013-602, State of Rio de Janeiro Brazil; ‡ 28109 Universidade Estadual do Norte Fluminense Darcy Ribeiro, Laboratório de Tecnologia de Alimentos, Campos do Goytacazes 28.013-602, State of Rio de Janeiro Brazil; § Universidade de São Paulo, Instituto de Química de São Carlos. Department of Chemistry and Molecular Physics, São Carlos 13.560-970, Brazil

## Abstract

The DPPH (2,2-diphenyl-1-picrylhydrazyl) assay is one of the most widely used methods for determining the radical scavenging capacity (RSC), yet discrepancies between spectrophotometric and electron paramagnetic resonance (EPR) measurements remain insufficiently addressed. In this study, simultaneous RSC measurements were performed by EPR and spectrophotometry to investigate the systematic differences between the methods. The results demonstrate that spectrophotometric measurements can underestimate RSC due to residual absorbance arising from neutralized DPPH and eventual decomposition products, leading to significant differences in IC_50_ and antioxidant activity index (AAI) values compared with EPR data. To overcome this limitation, three correction approaches are proposed, improving agreement with EPR results and eliminating statistical differences in IC_50_ and AAI values. These procedures were validated using phenolic standards and further evaluated for pigmented compounds using malvidin-3,5-diglucoside chloride and natural jambolan (*Syzygium cumini*) juice, an anthocyanin-rich sample. Although pigmentation affects high-concentration measurements, it does not significantly influence IC_50_ and AAI values after an appropriate absorbance correction. The proposed methodologies enhance the accuracy of the spectrophotometric DPPH assay and enable reliable comparison with EPR-based results, contributing to the improved standardization of RSC measurements.

## Graphical abstract

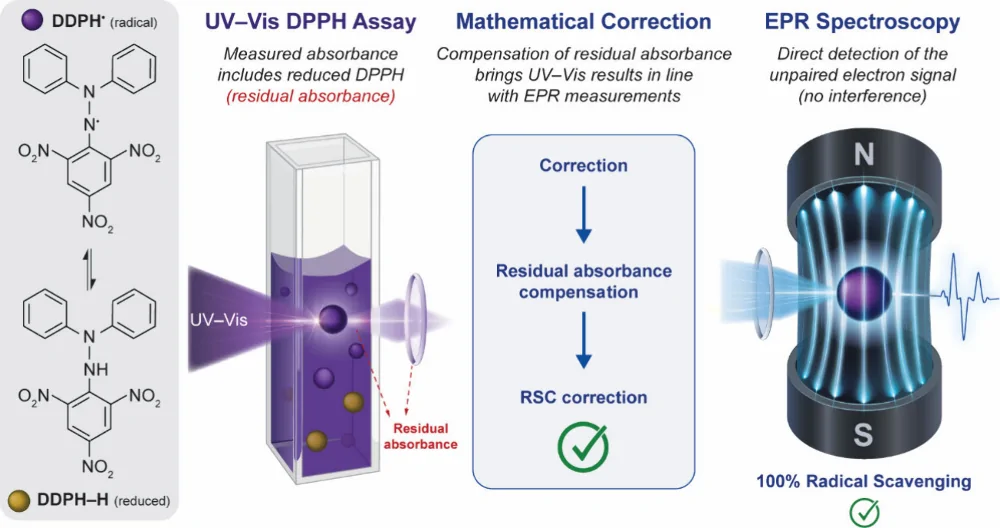

## Introduction

Free radicals and related reactive species have been extensively studied due to their significant biological and pathological implications.^1,2^ These species contain unpaired electrons and readily react with biomolecules, initiating chain reactions that generate additional reactive intermediates. In humans, reactive oxygen species (ROS) and reactive nitrogen species (RNS) are intrinsic by-products of cellular respiration and are continuously generated under physiological conditions. At basal concentrations, they function as signaling molecules that regulate various cellular processes, including cell proliferation, differentiation, and apoptosis.^3–5^ However, under pathological or adverse physicochemical conditions, excessive free radical production may overwhelm endogenous antioxidant defenses, ultimately resulting in cellular damage. Radical-mediated damage can oxidatively modify lipids, proteins, and DNA, processes that have been strongly associated with aging and various human diseases.^6^

In this context, antioxidants have attracted considerable scientific interest, since they can interact with free radicals, neutralizing them before critical biomolecules are damaged. In addition to endogenous antioxidants (enzymatic and non-enzymatic), accumulating evidence suggests that the intake of foods and natural products with antioxidant and other bioactive properties is associated with a reduced incidence of several chronic diseases.^7^ Although no single universal method can accurately assess antioxidant activity and underlying mechanisms, screening assays have been developed to identify potential antioxidant compounds. Several *in vitro* assays are widely used, including oxygen radical absorbance capacity (ORAC), ferric reducing antioxidant power (FRAP), cupric-ion reducing antioxidant capacity (CUPRAC), and radical scavenging assays based on 2,2-diphenyl-1-picrylhydrazyl (DPPH), and 2,2′-azino-bis­(3-ethylbenzothiazoline-6- sulfonic acid) (ABTS).^8–10^ Among these, the DPPH assay is the most frequently reported in the literature.

The use of DPPH as a method for the antioxidant assay in biological materials was first proposed by Blois in 1958.^11^ The method can be performed in both polar and nonpolar organic solvents and is based on the reduction of the DPPH radical, which results in a measurable color change. Upon reduction by an antioxidant, the deep violet DPPH radical is converted to its non-radical form, leading to a color change from violet to pale yellow. The stability of the DPPH solution and its strong absorbance band around 515 nm make it suitable for a straightforward and low-cost method. In addition to the conventional assay format, recent studies have expanded its applications. The method has been adapted for use in emulsions,^12^ for evaluating antioxidant interactions in binary mixtures,^13^ and for pre-column coupling with liquid chromatography–mass spectrometry, enabling the identification of individual antioxidant contributions.^14^ The DPPH radical scavenging capacity (RSC) is commonly expressed as the IC_50_ value, defined as the concentration of sample required to reduce the initial DPPH radical concentration by 50%. The IC_50_ is determined from a dose–response curve plotting the percentage of DPPH scavenged against the sample concentration. According to Scherer and Godoy,^15^ the antioxidant activity index (AAI) provides a more appropriate basis for comparing the scavenging capacity of different samples, given the lack of methodological standardization and the variability in initial DPPH concentrations and sample-to-reagent ratios reported in the literature. The AAI is calculated as the ratio of the initial DPPH concentration to the IC_50_ value, thereby minimizing the influence of assay conditions and improving comparability among studies.

However, two important questions arise: (i) In spectrophotometric measurements, it is observed that the RSC values reach a saturation value below 100%. Although this phenomenon has been reported in the literature,^16–18^ its potential to compromise the accuracy of the method has not been thoroughly discussed. (ii) What happens when the method is applied to colored natural extracts containing compounds whose absorption band overlaps with that of DPPH. A typical example is the presence of anthocyanins, a class of pigments responsible for the red, purple, and blue coloration of many fruits, flowers, and vegetables. Although interference from colored samples has been reported in the literature,^10^ no consensus has been reached regarding its impact on DPPH assay results. For instance,^19^ using Youden’s graphical method, concluded that colored fruit extracts do not affect the results. In contrast, other authors have applied the technique without addressing potential color interference.^20–23^ An alternative approach is electron paramagnetic resonance (EPR), which allows for direct access to the unpaired electron in the DPPH radical form. EPR is a sensitive technique widely used for detecting free radicals and other paramagnetic species.^24,25^ The intensity of the EPR signal is proportional to the number of unpaired spins, making the technique suitable for antioxidant evaluation using the DPPH assay,^26,27^ avoiding any interference from sample pigmentation. However, studies comparing spectrophotometry and EPR for the DPPH assay have reported significant differences in the RSC values obtained by the two methods. These discrepancies remain controversial, particularly because spectrophotometric measurements may be affected by sample pigmentation. Some authors attribute these differences to spectral interference caused by sample coloration,^28,29^ an effect not observed by Sanna et al.^30^ Overall, these studies suggest that EPR may provide a more reliable approach for DPPH-based antioxidant evaluation.

This work aims to explore and clarify the two questions concerning the spectrophotometric method described above. For that, we perform simultaneous measurements using UV-vis spectrophotometry and EPR for the determination of the DPPH radical scavenging capacity. Standard solutions of compounds exhibiting moderate to high antioxidant capacities were prepared over a broad concentration range. Scavenging response curves were constructed from the linear region to the saturation plateau, enabling a comparison of the techniques, particularly through statistical analysis of IC_50_ values. On the basis of the EPR measurements, we propose correction procedures for the spectrophotometric data, improving the accuracy and enhancing comparability between the two techniques. The influence of pigmentation on the spectrophotometric measurements was evaluated using malvidin 3,5-diglucoside chloride, as an anthocyanin standard, and natural jambolan (*Syzygium cumini*) juice, an anthocyanin-rich sample.^31,32^ Finally, we propose an experimental protocol to verify the existence of pigmentation interference and, if it exists, to eliminate its effects in order to obtain accurate results.

## Materials and Methodology

### Standards and Reagents

The free radical (DPPH) and the high-purity standard compounds, such as ellagic acid (CAS 476-66-4), l-ascorbic acid (CAS 50-81-7), (+)-catechin (CAS 154-23-4), gallic acid (CAS 149-91-7), caffeic acid (CAS 331-39-5), and malvidin 3,5-diglucoside chloride (CAS 16727-30-3) were purchased from Sigma-Aldrich. Ethanol (95%) was used as the solvent and was obtained from Synth.

### Jambolan Juice Preparation

Jambolan fruits were purchased from a local market (Campos dos Goytacazes, Rio de Janeiro, Brazil). The fruits were washed, sanitized, and frozen prior to pulp extraction. The pulp was extracted by expression using a commercial fruit extractor, as described by Mussi et al.,^33^ and centrifuged at 4,000 rpm for 10 min. The resulting supernatant was collected and stored at −20 °C as clarified juice until analyses.

Total monomeric anthocyanins content was determined by the pH-differential assay and expressed as cyanidin-3-glucoside equivalents in μg/mL of juice, according to Lee et al.^34^ The total phenolic content (TPC) was determined by the Folin-Ciocalteu assay^35^ and the results were expressed as μg of gallic acid equivalents (GAE) per mL of juice. The juice dry matter was determined by drying a 20.0 mL of sample aliquot to a constant weight in an oven at 105 °C, with results expressed as mg of solids per mL of juice. This dry matter content was used to calculate the concentrations of the various juice dilutions used in the antioxidant capacity measurements, expressed in mg of dried juice/mL. The clarified juice contained (303 ± 11) μg/mL of total anthocyanins, corresponding to (3.3 ± 0.1) μg/mg of dried juice, and (1070 ± 50) μg GAE/mL of total phenolics, corresponding to (11.6 ± 0.5) μg GAE/mg of dried juice.

### Antioxidant Capacity Measurements

The scavenging of DPPH in ethanolic solution (37.5 μg/mL) was used to evaluate the antioxidant capacity of standard solutions and clarified jambolan juice. Prior to the analysis, the juice was diluted with ultrapure water at various concentrations. Ethanolic solutions of each standard compound and diluted juice samples were prepared at different concentrations, mixed with DPPH in 2 mL vials, and stirred for 40 min prior to analysis, which was the time necessary to stabilization of the RSC, determined based on previous measurements with different samples (data not shown). The reaction mixtures were then measured simultaneously by EPR and spectrophotometry, with each measurement performed in triplicate. All reported concentrations of DPPH and samples correspond to the final concentrations in the reaction mixture.

EPR measurements were carried out at room temperature using a Bruker Elexsys E500 spectrometer equipped with a high-sensitivity cavity operating at X-band (∼9.8 GHz). The spectra were recorded with a magnetic field modulation frequency of 100 kHz, a modulation amplitude of 1 G, microwave power of 10 mW and a scan time of 60 s. Absorbance at 515 nm was measured using a kasuaki UV-Vis spectrophotometer (model V-1100PC).

The RSC values obtained by EPR and UV–Vis spectrophotometry were calculated according to the equation shown below:

$$RSC\left(\%\right)=100\times \frac{{I\left(A\right)}_{DPPH}-{I\left(A\right)}_{mix}}{{I\left(A\right)}_{DPPH}}$$ 1

where *I*(*A*)_*DPPH*_ is the EPR signal intensity (or absorbance, in the case of UV–Vis measurements) of the DPPH solution and *I*(*A*)_*mix*_ corresponds to the signal intensity (or absorbance) of the mixture of sample and DPPH. The following Equation was used to correct the RSC values obtained from the spectrophotometric method:

$${RSC}_{corrected}\left(\%\right)=100\times \frac{A_{DPPH}-\left(A_{mix}-(\frac{RSC}{100})\times A_{Res}\right)}{A_{DPPH}}$$ 2

where *A*_*Res*_ is the residual absorbance, measured for a mixture in which DPPH is completely neutralized, and the *RSC* value may be obtained from either EPR or spectrophotometric measurements, as discussed in the Results and Discussion.

### Statistical Analyses and Data Treatment

All analyses were performed at least in triplicate, and values were expressed as mean ± standard deviation. The IC_50_ and AAI values were subjected to analysis of variance (ANOVA) and Tukey test.^36^ The statistical analyses were performed using R software^37^ through RStudio.^38^ Differences were considered statistically significant at the 95% confidence level (p ≤ 0.05). EPR spectrum simulations were carried out using the EasySpin package for MATLAB (The MathWorks, Inc).

## Results and Discussion

### Non-Pigmented Standard Compounds

We first present the measured quantities obtained from both the analytical methods. In Figure 1a, the experimental EPR data (magenta) are superimposed to the simulated spectrum (black). The five-line pattern, with a relative intensity ratio of 1:2:3:2:1, arises from hyperfine interactions with two nearly equivalent nitrogen nuclei and is characteristic of DPPH in solution. The values obtained from the simulation for the g values and the hyperfine splitting constants are g = 2.0035, a_N1_ = 6.76 G, and a_N2_ = 6.34 G, following the literature.^25^ Because continuous-wave EPR detects the first derivative of the absorption signal due to magnetic field modulation for phase-sensitive detection, the absorption spectrum (inset in Figure 1a) corresponds to the integral of the recorded EPR spectrum. Thus, strictly speaking, the EPR intensity is obtained by double integration of the spectrum, meaning the area under the absorption curve. This can be performed using the simulated curve or by numerical integration of the experimental data. For sample concentrations with high RSC values (*i*.*e*., low concentration of DPPH radicals), the EPR signal is reduced bringing considerable uncertainty to the numerical integral. On the other hand, as simulating many curves (several concentrations in triplicate) becomes time-consuming, the peak-to-peak value of the central line is considered as an alternative procedure for the EPR intensity determination.^30,39^ For the spectrophotometer method, the absorbance is measured at 515 nm, where the radical DPPH presents maximum absorbance (Figure 1b, in magenta).

**Figure 1:**
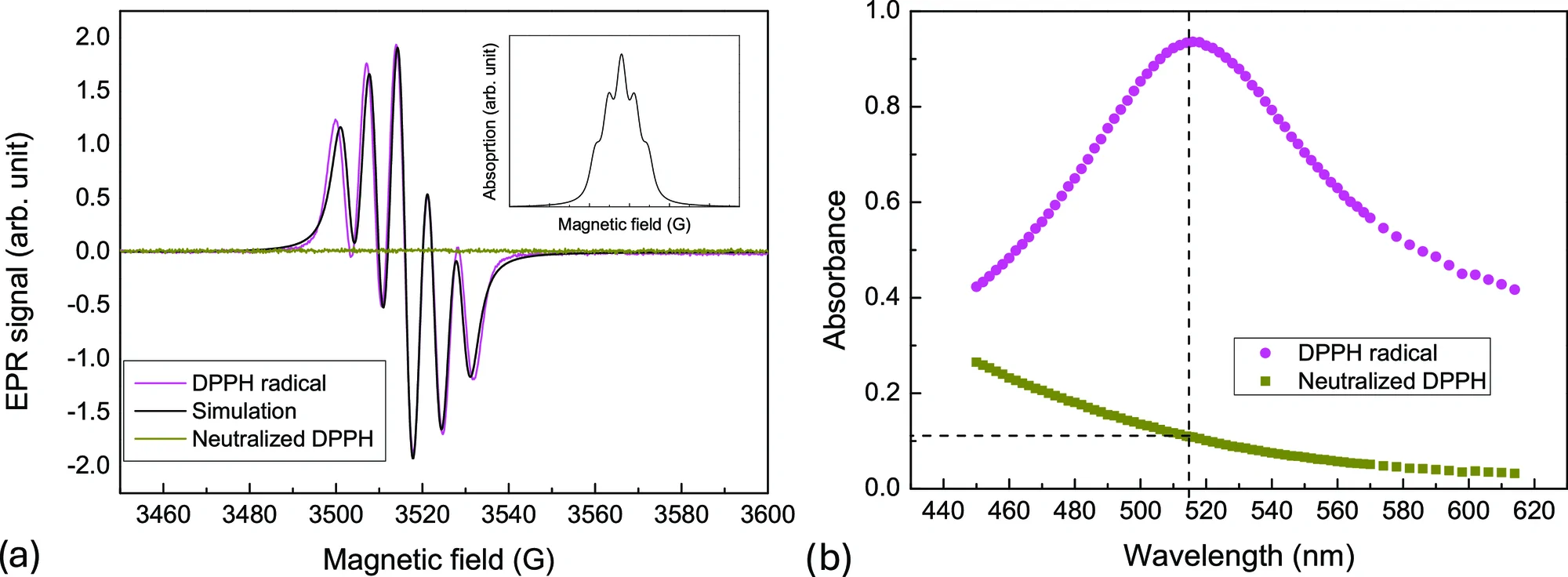
(a) EPR spectra (magenta) and simulation (black) for DPPH radical in solution, and EPR spectra (dark yellow) for neutralized DPPH. The inset shows the absorption curve (first integral). (b) Absorbance spectra for DPPH radical (magenta) and neutralized DPPH (dark yellow).

Figure 2 shows the proof of principle of the methods, evidencing the linearity of both EPR intensity and absorbance with DPPH concentration. Besides, the line width of the spectra is not affected by the antioxidants (Figure S1). The spectrophotometer method presents a linearity limit (around 75 μg/mL), whereas the EPR exhibits linearity throughout the entire range.

**Figure 2:**
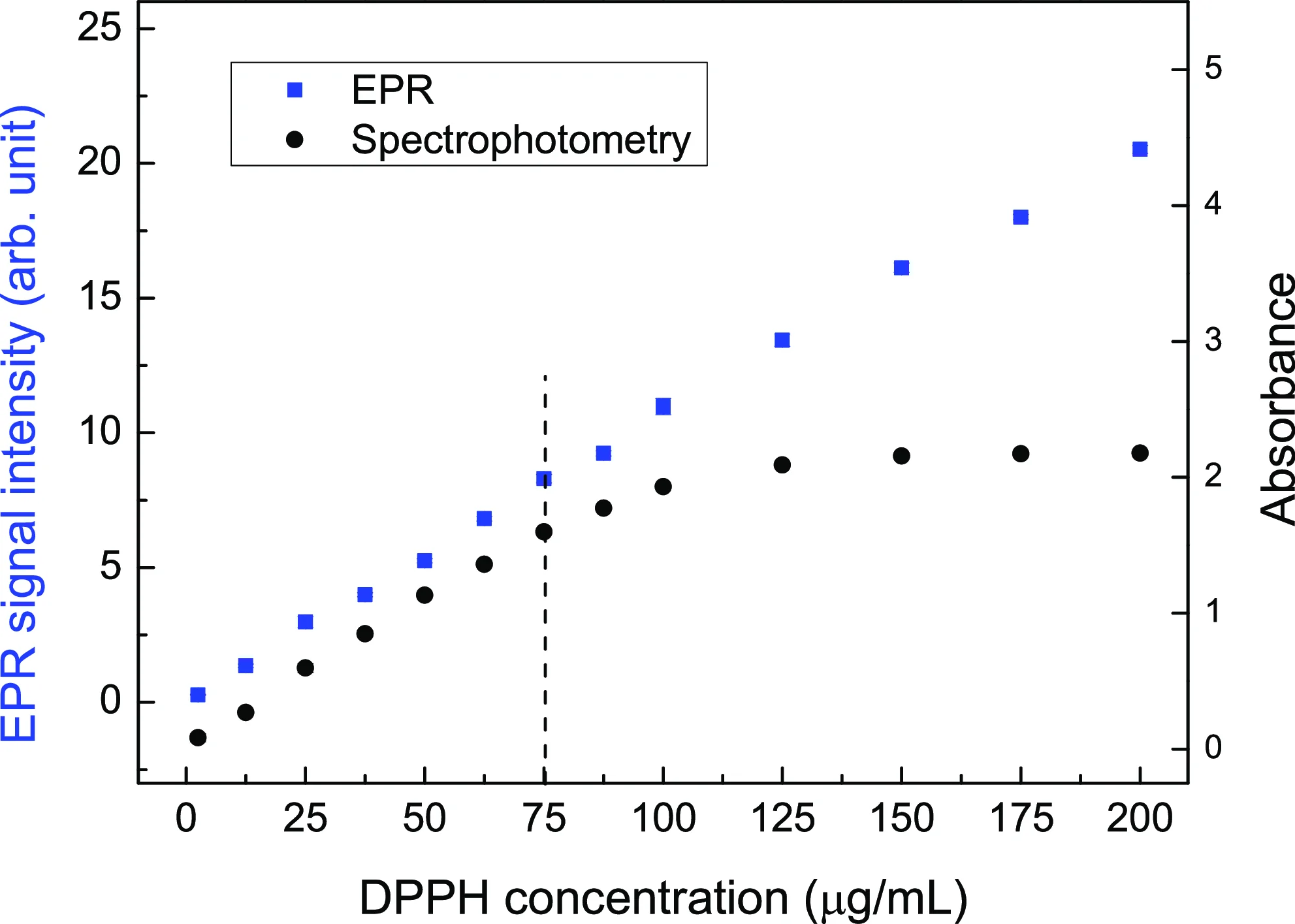
Absorbance and EPR intensity *versus* DPPH concentration.

Figure 3 and Table 1 present the results obtained for ellagic acid as a representative sample, comparing the RSC curves determined by both methods and the corresponding IC_50_ values. Figure 3a shows RSC as a function of sample concentration, measured by EPR (blue) and spectrophotometry (black). In both cases, the RSC increases linearly at low concentrations before reaching saturation. The plotted curves represent the mean RSC values. For statistical analysis and determination of IC_50_ values and their associated errors, linear regression was performed within the linear range for each independent triplicate experiment. The two methods clearly diverged at higher concentrations. For EPR, the maximum response reaches a RSC of 100%, whereas this is not observed for the spectrophotometric method, which reaches approximately 70% for ellagic acid (Figure 3a). Most papers consider only the linear ranges of the curves. Nevertheless, saturation below 100% in spectrophotometric measurements has been reported in the literature,^13,40,41^ although its underlying cause is rarely discussed. To rationalize this divergence, we refer back to Figure 1. At high sample concentrations, when DPPH is totally neutralized, the EPR signal vanishes (Figure 1a, in dark yellow) resulting in a RSC of 100% according to eq 1. In contrast, for the spectrometric measurements, although the characteristic absorption band of the DPPH radical disappears, a residual absorbance (*A*_*Res*_) remains at 515 nm, as remarked by dashed lines in Figure 1b. This residual signal leads to an underestimation of RSC when calculated using eq 1. In the saturation region, a correction could be done by just taring the spectrophotometer baseline or subtracting *A*_*res*_ from *A*_*mix*_ during data analysis processing. However, such procedures would not be valid within the linear range of interest, where a fraction of DPPH remains in the radical form, while another fraction is neutralized.

**Figure 3:**
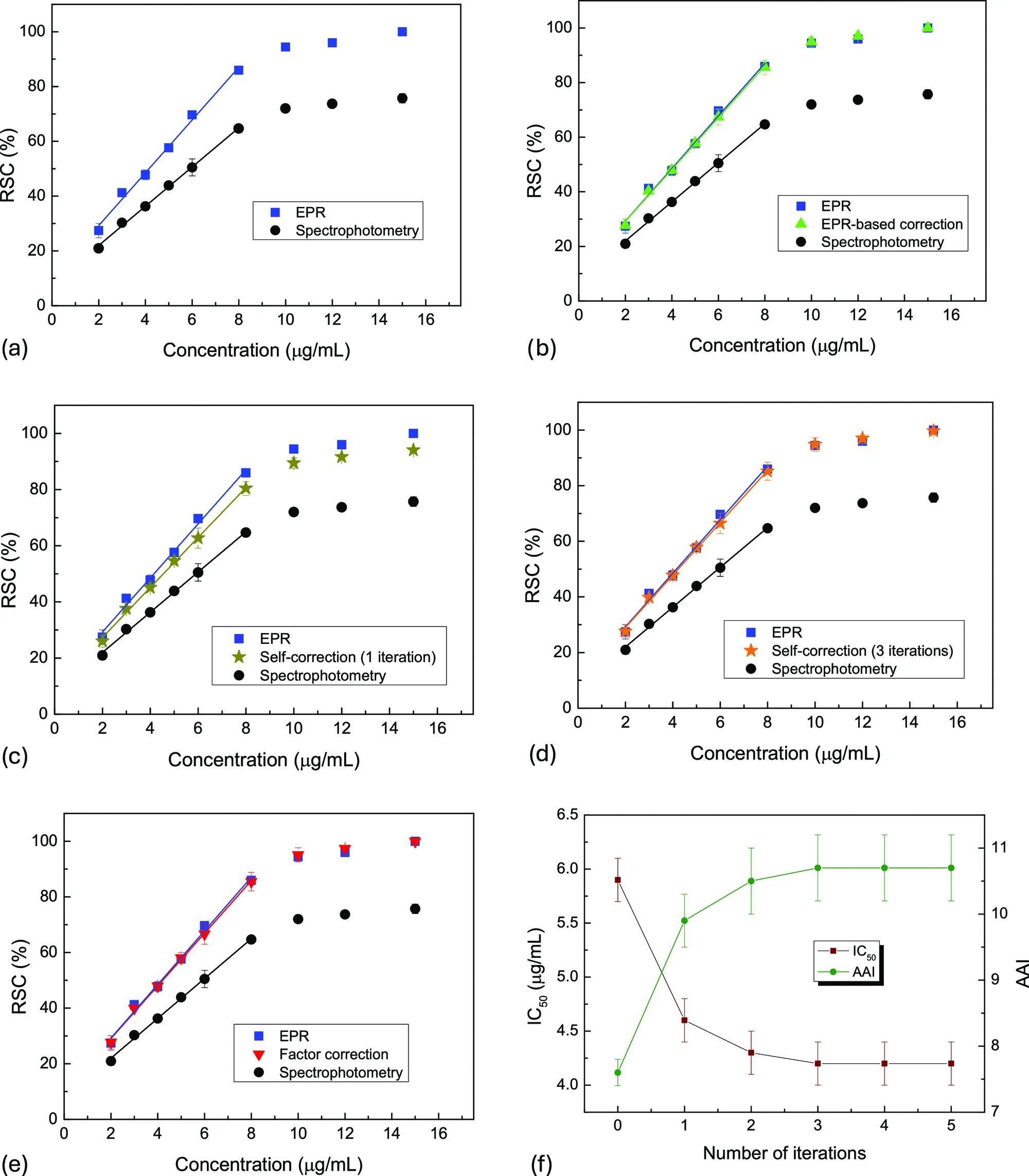
(a) RSC measured by EPR (blue) and spectrophotometry (black) for ellagic acid and the proposed (b) EPR-based correction, (c, d) self-correction and (e) factor correction. (f) IC_50_ and AAI *versus* number of iterations for the self-correction approach.

**Table 1:** IC_50_ and AAI Obtained from EPR, Spectrophotometry and after Different Corrections Using Ellagic Acid as a Representative Sample^a^

	*IC*_50_ (μg/mL)	*AAI*
Spectrophotometry	5.9 ± 0.2^*a*^	7.6 ± 0.2^*c*^
EPR-based correction	4.2 ± 0.2^*c*^	10.7 ± 0.5^*a*^
Self-correction (1 iteration)	4.6 ± 0.2^*b*^	9.9 ± 0.4^*b*^
Self-correction (3 iteration)	4.2 ± 0.2^*c*^	10.6 ± 0.6^*a*^
Self-correction (5 iteration)	4.2 ± 0.2^*c*^	10.7 ± 0.6^*a*^
Factor correction	4.2 ± 0.2^*c*^	10.7 ± 0.6^*a*^
EPR	4.2 ± 0.1^*c*^	10.8 ± 0.3^*a*^

a Values within a column with the same letter show no significant differences.

Here, we propose three different approaches to correcting the spectrophotometric data.

#### (i) EPR-Based Correction

For the saturation range (complete DPPH neutralization), in addition to baseline correction, *A*_*Res*_ may be subtracted from *A*_*mix*_ during data processing. However, for a given sample concentration within the linear range, the RSC value reflects only the fraction of DPPH that has been neutralized. Therefore, directly subtracting *A*_*Res*_ from *A*_*mix*_ is not appropriate, as the residual absorbance should be proportional to the neutralized fraction rather than treated as a constant offset. To account for this, we propose the use of eq 2, where the *A*_*Res*_ is weighted by the RSC value obtained from EPR measurements. The simultaneous acquisition of EPR and spectrophotometric data strengthens the validity of this procedure. The corrected spectrophotometric curve (Figure 3b) closely matches the EPR curve, and both IC_50_ and AAI showed no statistically significant differences (Table 1) (p ≥ 0.05).

#### (ii) Self-Correction

Unless the work aims to compare EPR and spectrophotometric methods for RSC measurements, researchers generally select only one technique. Thus, when only the spectrophotometric method is employed, an iterative correction procedure is proposed. In the first approximation, the RSC values obtained using eq 1, are used as input in eq 2. As shown in Figure 3c, in the first iteration, the values approximate those measured by EPR, but still with significant differences when comparing IC_50_ and AAI (Table 1). The new RSC value thus serves as input for the next iteration and so on. Figure 3d shows that the values match the EPR curve after three iterations. A clear depiction of the iterative procedure is illustrated in Figure 3e, where both IC_50_ and AAI values stabilize after three iterations, showing no statistically significant differences when compared to the EPR measurements (Table 1, p ≥ 0.05).

#### (iii) Factor Correction

Finally, we present a simple empirical method for correcting the spectrophotometric RSC values, in which all the RSC values obtained by spectrophotometry are multiplied by a constant factor *F*_*corr*_ = 100/*RSC*_*SAT*_, where *RSC*_*SAT*_ is the RSC value in the saturation range. Similarly to the previous correction procedures, the corrected curve shows strong agreement with the EPR curve, and IC_50_ and AAI values present no statistically significant differences relative to the EPR measurements (Table 1, p ≥ 0.05).

In addition to ellagic acid, other phenolic standards were investigated to confirm the residual absorbance effect in spectrophotometric measurements and to validate the proposed correction procedure. Simultaneous RSC measurements were performed for ascorbic acid, catechin, gallic acid, and caffeic acids. The divergence between the EPR and spectrophotometric curves was observed for all samples (Figure S2), and it was more pronounced for some samples than for others. The RSC saturation values measured by the spectrophotometer ranged from 76% to 92%, meaning that the residual absorbance differs from one sample to another. These differences are visually evident.

Figure 4 illustrates that samples with completely neutralized DPPH showed different colors. Ellagic acid and catechin turned red-brown, whereas caffeic, ascorbic, and gallic acids turned yellow. This observation may be related to the antioxidant mechanisms involved. The action of antioxidants can be classified into two neutralization mechanisms: single electron transfer (SET) and hydrogen atom transfer (HAT), in which the radical (DPPH, in this case) can receive one electron or one proton, respectively. Phenolic compounds can undergo both mechanisms,^42^ and DPPH in solution acquires a red-brown color for SET and a yellow color for HAT.^43^ As discussed for ellagic acid, the effect of the residual absorbance is not only on the saturation values but also on the linear range of the data (Figure S2). The IC_50_ and, consequently, AAI values significantly differ between the EPR and spectrophotometric methods for all samples. After correcting the curves (using *F*_*corr*_), the values no longer show significant differences (Table 2), thus validating the procedure. Decomposition products can also contribute to the residual absorbance,^44^ but as they are produced proportionally to the neutralized radicals, the proposed corrections inherently account for them and remain valid.

**Figure 4:**
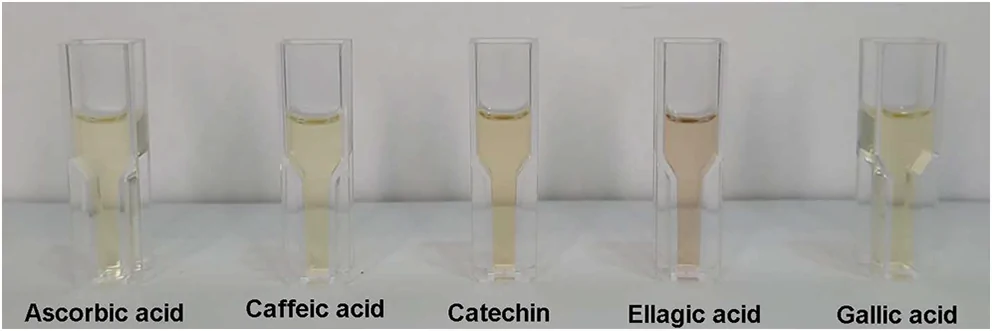
Pictures of non-pigmented standard compound samples with completely neutralized DPPH.

**Table 2:** IC_50_ and AAI Obtained from EPR, Spectrophotometry and after Factor Correction for Uncolored Standard Compounds^a^

	*IC*_50_ (μg/mL)			*AAI*		
	Spectro	Factor correction	EPR	Spectro	Factor correction	EPR
Ellagic acid	5.9 ± 0.2^*a*^	4.2 ± 0.2^*c*^	4.2 ± 0.1^*c*^	7.6 ± 0.2^*c*^	10.7 ± 0.6^*a*^	10.8 ± 0.3^*a*^
Ascorbic acid	4.7 ± 0.1^*a*^	4.0 ± 0.1^*b*^	4.04 ± 0.07^*b*^	8.0 ± 0.2^*b*^	9.4 ± 0.3^*a*^	9.3 ± 0.2^*a*^
Catechin	4.9 ± 0.1^*a*^	4.00 ± 0.09^*b*^	3.84 ± 0.09^*b*^	7.6 ± 0.2^*b*^	9.4 ± 0.2^*a*^	9.7 ± 0.2^*a*^
Gallic acid	1.46 ± 0.05^*a*^	1.33 ± 0.05^*b*^	1.32 ± 0.04^*b*^	25.7 ± 0.9^*b*^	28 ± 1^*a*^	28.5 ± 0.8^*a*^
Cafeic acid	3.03 ± 0.04^*a*^	2.79 ± 0.03^*b*^	2.5 ± 0.1^*b*^	12.4 ± 0.2^*b*^	13.4 ± 0.2^*b*^	14.9 ± 0.2^*a*^

a Values within a line with the same letter present no significant differences.

It is worth synthesizing the core aspects of the corrections presented above. The motivation for the proposed corrections stems from the fact that spectrophotometry is the simplest, fastest, and most cost-effective method for the DPPH assay, whereas EPR yields the most reliable RSC values and is, therefore, used as the reference standard. The EPR correction (i) has physical principles, with the absorbance being weighted by RSC from EPR, so that its results validate the use of eq 2. The self-correction (ii) is based on the same physical principles, but as the RSC values from the spectrophotometer are underestimated, an iterative procedure is necessary. Factor correction (iii) is an empirical and simpler approach. The RSC values in the saturation range are forced to reach 100%, with data out of the saturation proportionally increasing. In practical terms, for the spectrophotometric-based assay, either self (ii) or factor (iii) corrections can be used. Correction factor (iii) offers the advantage of a single mathematical step, although both approaches give similar results. Besides improving the accuracy of the spectrophotometric method, data correction enables a comparison of the IC_50_ and AAI values obtained with this method to those reported in the literature using EPR.

### Pigmented Standard Samples

The samples analyzed so far are colorless, and the divergence between EPR and spectrophotometric data concerns only the residual absorbance of neutralized DPPH and the eventual decomposition products. To assess the influence of sample pigmentation, we report results for malvidin 3,5-diglucoside chloride, a type of anthocyanin, and jambolan juice as a real anthocyanin-rich sample. For malvidin, Figure 5a shows that, for the spectrophotometric data (black symbols), the data maintain the linear behavior for low concentrations, but the RSC values do not saturate at high sample concentrations, which is clearly influenced by pigmentation. In this range, DPPH is completely neutralized, but the absorbance measured due to the pigment underestimates the RSC value when using eq 1. It is possible to correct the curve by subtracting the sample absorbance (orange symbols) from the data before applying eq 1. The corrected curve, in magenta, behaves like those observed for the colorless samples, saturating below 100%. The values for IC_50_ and AAI (Table 3) do not differ statistically, which is explained by the negligible absorbance values of the sample (orange symbols) in the linear range of RSC. After this pigment correction, we apply the factor correction (*F*_*corr*_) (red symbols) so that the curve matches the EPR curve (blue symbols), and the values for IC_50_ and AAI are statistically equal to those obtained by the EPR.

**Figure 5:**
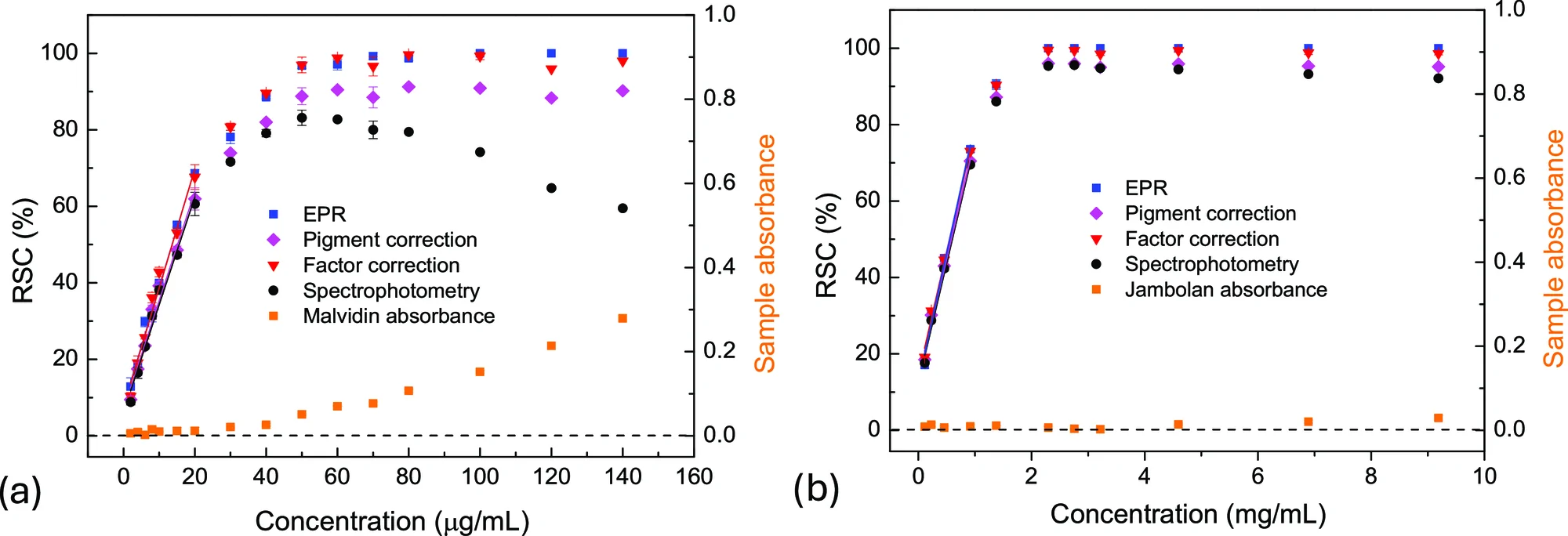
Pigmented samples: EPR (blue) and spectrophotometry (black) RSC measurements, sample absorbance (orange), with pigment (magenta) and factor (red) corrections for (a) malvidin and (b) jambolan. The concentration in (b) refers to mass of solids of juice per mL.

**Table 3:** IC_50_ and AAI Obtained from EPR, Spectrophotometry and after Pigment and Factor Corrections for Malvidin and Jambolan^a^

	Malvidin		Jambolan	
	*IC*_50_ (μg/mL)	*AAI*	*IC*_50_ (mg/mL)^b^	*AAI*
Spectrophotometry	15.6 ± 0.5^*a*^	2.9 ± 0.1^*b*^	0.595 ± 0.005^*a*^	0.0588 ± 0.0005^*a*^
Pigment correction	15.2 ± 0.6^*a*^	2.9 ± 0.1^*b*^	0.591 ± 0.006^*a*^	0.0592 ± 0.0006^*a*^
Factor correction	13.4 ± 0.5^*b*^	3.3 ± 0.1^*a*^	0.555 ± 0.006^*b*^	0.0631 ± 0.0007^*b*^
EPR	13.6 ± 0.2^*b*^	3.30 ± 0.06^*a*^	0.556 ± 0.005^*b*^	0.0629 ± 0.0006^*b*^

a Values within a column with the same letter present no significant differences.

b The jambolan concentration refers to the mass of solids of juice per mL.

For natural samples extracted from fruits or vegetables in shades of purple, the influence is expected to be even smaller, since there are many other antioxidant compounds.^19^ For jambolan juice, as a case study, Figure 5b shows that the pigmentation slightly influenced the RSC values (black symbols) for high sample concentrations, as the sample absorbance is very small even in this range (orange symbols). Similar to malvidin, the jambolan pigmentation did not affect the IC_50_ and the AAI values significantly, and further factor correction (*F*_*corr*_) (red symbols) matches the data to the EPR curve (blue symbols), with statistically equal values for IC_50_ and AAI (Table 3), just like for the other samples. Therefore, to ensure reliable results for pigmented samples, their absorbance can be measured in each case to correct for the RSC data.

## Conclusions

This study clarifies two overlooked aspects in the literature, namely, the underestimation of RSC values and the confounding effects of sample pigmentation on results obtained via spectrophotometric DPPH assays.

Briefly, our simultaneous measurements reveal a clear distinction between the EPR and spectrophotometric assays. EPR provides superior accuracy as it directly measures the free radical concentration without any residual signal. The DPPH assay by the spectrophotometric method underestimates the RSC values because of the residual absorbance of completely neutralized radicals and eventual decomposition products. The validity of correcting the spectrophotometric RSC accounting for the residual absorbance is confirmed by EPR correction (i), which utilizes EPR data. Relying solely on the spectrophotometric method, self-correction (ii) is proposed, which incorporates residual absorbance correction through an iterative process. Although this approach is effective and based on physical concepts, the iterative approach is not straightforward. As an alternative, factor correction (iii) offers an empirical approach through a significantly more straightforward procedure, providing a practical tool for routine analysis. Besides improving the accuracy of the spectrophotometric method, data correction enables a comparison of results obtained with this method to those reported in the literature using EPR. For further studies, chemical analyses can provide relevant information so that the residual absorbance allows one to infer which neutralization mechanism (SET or HAT) is predominant in a specific assay.

Moreover, concerning pigmented samples, we believe that the findings address potential concerns regarding pigment interference in the spectrophotometric method. As a case study, we showed that the pigment interference of the pure standard malvidin 3,5-diglucoside chloride and jambolan juice in the spectrophotometric RSC measurement occurs at high sample concentration, when DPPH is completely neutralized, but it does not affect the IC_50_ and AAI values significantly. For other samples extracted from fruits or vegetables in shades of purple, the influence is expected to be small since there are many other antioxidant compounds. Nevertheless, when pigment interference is suspected, before the correction (i, ii, or (iii) discussed above, this interference can be eliminated by subtracting the sample absorbance, and the results can be properly analyzed.

## Supplementary Material



## Notes

Assessment of the line width of the DPPH spectra under different sample conditions and RSC curves from EPR and spectrophotometry for several non-pigmented standard compounds (PDF)
