# Supplementary material for: Comparative Evaluation of Electron Paramagnetic Resonance and Spectrophotometry for DPPH-Based Antioxidant Capacity Determination
Source: ACS Omega. 2026 Sep 22;11(39):58800–8. doi: 10.1021/acsomega.6c03248 (PMC13647961; doi:10.1021/acsomega.6c03248)
Supplement: Supplementary file 1 [file ao6c03248_si_001.pdf]

Comparative evaluation of electron paramagnetic resonance and spectrophotometry for DPPH-based antioxidant capacity determination

[1]Helvis L. S. Fontoura, [1]Ana Flávia L. M. Nascimento, [1]Francine A. F. Menezes, [2]Pâmela V. Rocha, [3]Stanislau Bogusz Jr., [2]Nádia R. Pereira, *[1]André O. Guimarães

[1] *Universidade Estadual do Norte Fluminense Darcy Ribeiro (UENF), Laboratório de Ciências Físicas, Campos do Goytacazes, RJ, Brazil*

[2] *Universidade Estadual do Norte Fluminense Darcy Ribeiro (UENF), Laboratório de Tecnologia de Alimentos, Campos do Goytacazes, RJ, Brazil*

[3] *University of São Paulo (USP), São Carlos Institute of Chemistry (IQSC), São Carlos, São Paulo, SP, Brazil.*

SUPPORTING INFORMATION

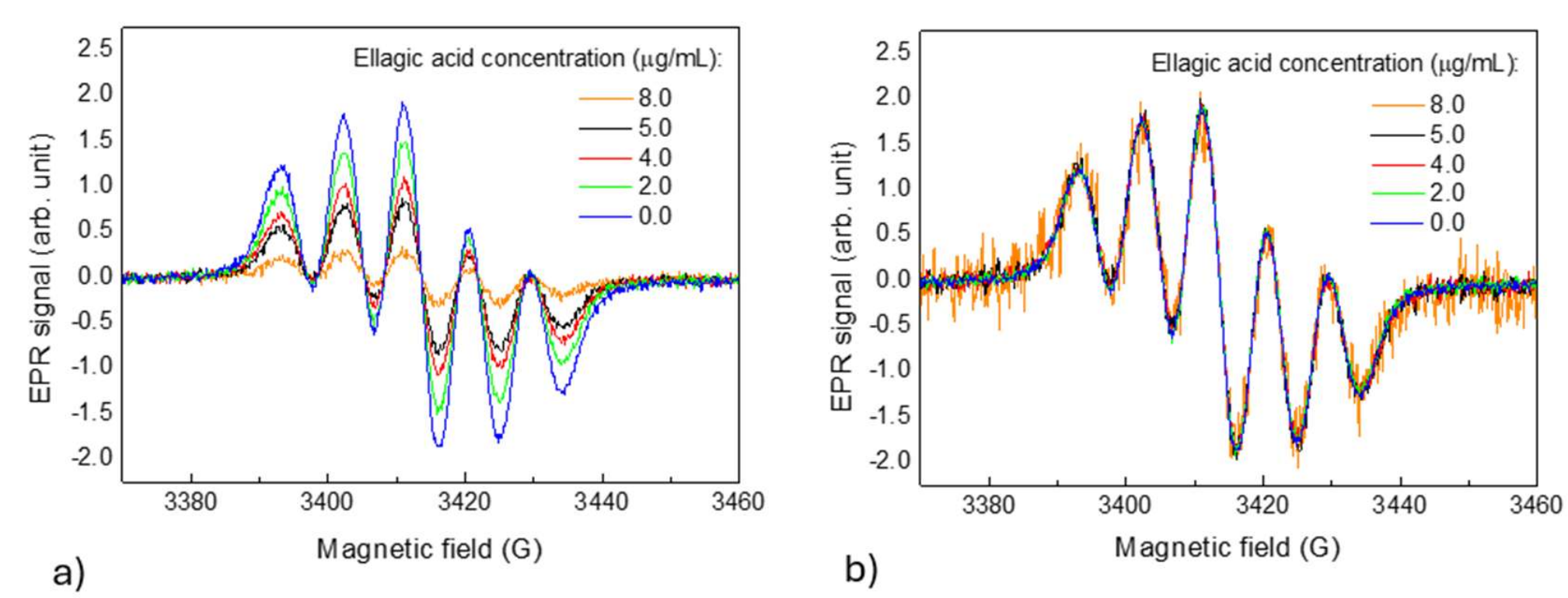

Figure S1: Linewidth assessment: (a) As measured and (b) normalized EPR spectra for DPPH solutions with different ellagic acid concentrations.

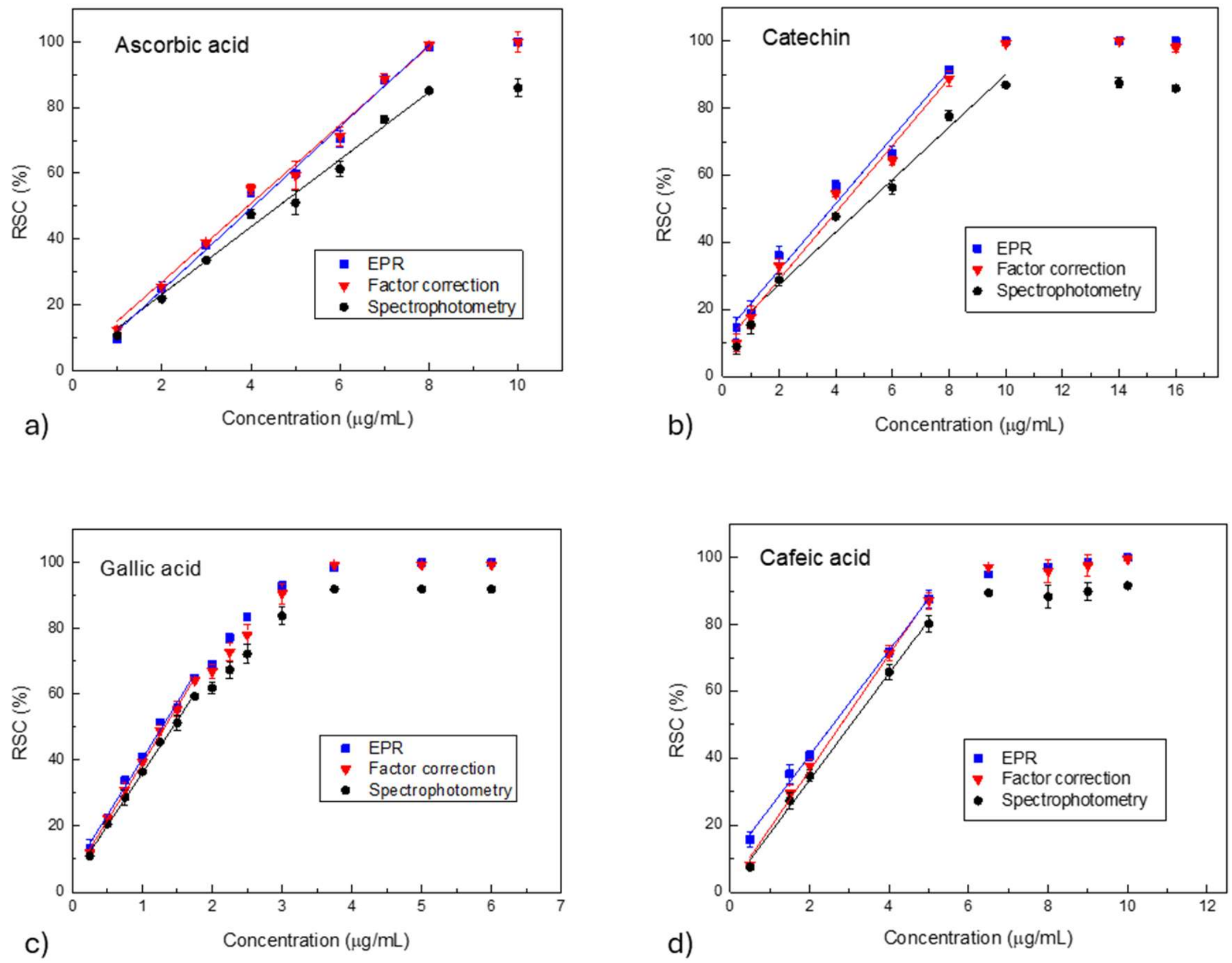

Figure S2: Data from EPR (blue), spectrophotometry (black) and factor correction (red) for non-pigmented standard compounds: (a) Ascorbic acid, (b) catechin, (c) gallic acid and (d) caffeic acid.
